# Discovery of Sulforaphane as a Potent BACE1 Inhibitor Based on Kinetics and Computational Studies

**DOI:** 10.3390/nu12103026

**Published:** 2020-10-02

**Authors:** Kumju Youn, Jeong-Hyun Yoon, Nayoung Lee, Gyutae Lim, Jinhyuk Lee, Shengmin Sang, Chi-Tang Ho, Mira Jun

**Affiliations:** 1 Department of Food Science and Nutrition, College of Health Sciences, Dong-A University, Busan 49315, Korea; kjyoun@dau.ac.kr (K.Y.); yjhyun1110@donga.ac.kr (J.-H.Y.); nylee0420@donga.ac.kr (N.L.); 2 Center for Silver-Targeted Biomaterials, Brain Busan 21 Plus Program, Graduate School Dong-A University, Busan 49315, Korea; 3 Graduate Korean Bioinformation Center, Korea Research Institute of Bioscience and Biotechnology (KRIBB), Daejeon 34141, Korea; gyutae@kribb.re.kr (G.L.); jinhyuk@kribb.re.kr (J.L.); 4 Department of Bioinformatics, KRIBB School of Bioscience, Korea University of Sciences and Technology, Daejeon 34113, Korea; 5 Center for Excellence in Post-Harvest Technologies, North Carolina Agricultural and Technical State University, 500 Laureate Way, Kannapolis, NC 28081, USA; ssang@ncat.edu; 6 Department of Food Science, Rutgers University, New Brunswick, NJ 08901, USA; ho@aesop.rutgers.edu

**Keywords:** Alzheimer’s disease (AD), BACE1, in silico docking, sulforaphane

## Abstract

BACE1 is the rate-limiting enzyme involved in the production and deposition of β-amyloid (Aβ). Since neurotoxic Aβ plays a critical role in Alzheimer’s disease (AD) pathogenesis, BACE1 has emerged as a key target for preventing AD. In the present study, the potential of sulforaphane, an isothiocyanate found in cruciferous vegetables, as a BACE1 inhibitor has been investigated. Sulforaphane exhibited six times more potent activity against BACE1 compared to well-known positive controls including resveratrol and quercetin. Sulforaphane presented selective and non-competitive BACE1 inhibitory activity with low off-target inhibition of BACE2 and other aspartic and serine proteases. In addition, sulforaphane presented negative binding energy, suggesting that the compound had a high affinity for BACE1. It interacted with locations other than the active binding sites of BACE1 through van der Waals forces. Overall, sulforaphane appeared to be a promising candidate with potent and selective BACE1 inhibitory properties that play an important role in AD prevention.

## 1. Introduction

Alzheimer’s disease (AD) is a neurodegenerative disease characterized by cognitive declines and memory impairments. AD is the second most common cause of death among those aged 70 and older worldwide [1]. The dysregulation of the amyloid-beta (Aβ) peptide level results in senile plaques composed of aggregated Aβ, ultimately leading to neuronal death. Aβ, the primary factor initiating the pathological event in AD, is derived from the successive cleavage of amyloid precursor protein (APP) by β-and γ-secretase in the brain [2].

β-site amyloid precursor protein cleaving enzyme 1 (BACE1), the main form of β-secretase, is a membrane-bound aspartyl protease expressed mainly in the central nervous system (CNS). In addition, BACE1 is the rate-limiting enzyme responsible for the production of Aβ from APP and both the expression level and activity of this enzyme are aberrantly elevated in the brains of patients with AD [3]. It has been demonstrated that BACE1 gene deletion leads to the inhibition of Aβ accumulation and improvement of cognitive impairments in APP transgenic mice [4,5]. In contrast, achieving the reduction of Aβ levels by γ-secretase inhibitor was associated with numerous side effects such as skin cancer, gastrointestinal bleeding, and autoimmune issues due to concomitant changes in Notch signaling [6]. Therefore, BACE1 is a primary therapeutic target being currently investigated for AD-modifying intervention.

Sulforaphane (isothiocyanato-4-(methylsulfinyl)-butane) is a sulfur-rich compound found in cruciferous vegetables including broccoli and cabbage. Sulforaphane is not only a well-known chemoprotective compound but also demonstrated to have a variety of biological activities including antioxidant, antidiabetic, anti-inflammatory, and neuroprotective effects [7,8,9,10,11]. Recently, sulforaphane has attracted attention for improving cognitive impairments associated with Aβ toxicity and/or deposition in mouse models [12,13,14]. However, its direct effect on BACE1, the initiating and rate-limiting enzyme in Aβ production, has never been elucidated. Therefore, the present study aimed to evaluate the direct inhibitory effect and inhibition mode of sulforaphane against BACE1. In addition, molecular docking simulation was used to analyze whether the compound can reach the target enzyme to produce the biological effect safely and interact with the targeted sites.

## 2. Materials and Methods

### 2.1. Reagent

Sulforaphane (≥98% purity), resveratrol, and quercetin were purchased from Sigma-Aldrich (St. Louis, MO, USA). The aspartic proteases such as pepsin, cathepsin D, and their substrates were also purchased from Sigma-Aldrich (St. Louis, MO, USA). BACE1 fluorescence resonance energy transfer (FRET) kit was obtained from Thermo Fisher Scientific, Inc. (Waltham, MA, USA). BACE2 and synthetic peptide substrate were purchased from Enzo Life Sciences (Farmingdale, NY, USA).

### 2.2. Evaluation of BACE1 Activity, Kinetics, and Enzyme Selectivity

The fluorescence resonance energy transfer (FRET) assay for BACE1 and BACE2 was conducted following the supplier’s instructions, with minor modifications [15]. Briefly, a solution of BACE1 or BACE2 (1.0 U/mL), the substrate, and sulforaphane was incubated for 60 min at 25 °C in 96-well black plates. The increase in fluorescence was measured by a spectrofluorometer (FLX800, Winooski, VT, USA). The inhibitory activity (%) was obtained using the formula: Inhibition (%) = [1 − (S − S0)/(C − C0)] × 100, where C is the absorbance of control after 60 min of incubation, C0 is the absorbance of control at time 0, S is the absorbance of samples after 60 min of incubation, and S0 is the absorbance of the samples at time 0.

Inhibition mode and kinetic parameters including Ki, Km, and Vmax were evaluated by Dixon plot and Lineweaver–Burk plot. The Dixon plot was acquired in the presence of various substrate concentrations, which was intended to determine the mode of enzyme inhibition and Ki value for the enzyme–inhibitor complex. The Lineweaver–Burk plot was used to calculate Vmax and Km and from the absence or presence of sulforaphane (final concentration of 0.03, 0.3, 1.5, and 3.0 µM). SigmaPlot™ was used to analyze enzyme kinetics.

To evaluate enzyme selectivity, aspartic proteases including pepsin and cathepsin D were assessed with their substrates. In addition, serine proteases such as trypsin, chymotrypsin, and elastase assays were conducted.

### 2.3. In Silico Docking Simulation

Docking analysis was conducted on the conformation of the BACE1-sulforaphane with Autodock Vina program for predicting binding free energy and poses (version 1.1.2, The Scripps Research Institute, La Jolla, CA, USA). Pck software was employed to find the binding pocket residues of enzymes. The default box size was set to 30 Å × 30 Å × 30 Å. The structures of BACE1 and sulforaphane were retrieved from the Protein Data Bank (PDB ID: 2wjo) and PubChem (CID: 5350), respectively.

### 2.4. Statistical Analysis

All data were presented as the mean ± standard deviation (SD) of three independent experiments. Statistical significance was determined by Duncan’s multiple range tests using the SAS system (version 9.3, Cary, NC, USA).

## 3. Results and Discussion

### 3.1. BACE1 Inhibitory Activity, and Kinetic Parameters of Sulforaphane

**Figure 1:**
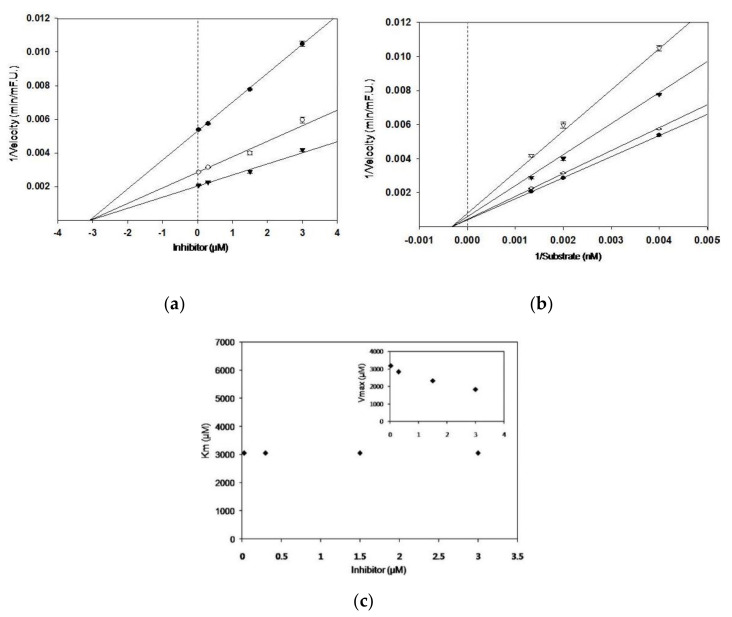
Dixon and Lineweaver–Burk plots for the inhibitory activity of sulforaphane against BACE1. Dixon plots show the effects of the presence of different substrate concentrations: -- 250 µM; -○- 500 µM; -▼- 750 µM for sulforaphane (**a**). Lineweaver–Burk plot was analyzed in the presence of different inhibitor concentrations: -●- 0.03 μM; -○- 0.3 μM; -▼- 1.5 μM; -▽- 3 μM for sulforaphane (**b**). The Km values as a function of the concentration of sulforaphane (Inset); dependence of the values of Vmax on the concentration of sulforaphane (**c**).

As shown in Table 1, sulforaphane significantly inhibited BACE1 in a concentration-dependent manner, from 0.03 to 3 μM, with IC_50_ value of 2.80 ± 0.19 μM. According to the Dixon and Lineweaver–Burk plots, sulforaphane suppressed BACE1 in non-competitive mode with a Ki value of 3.1 μM, which demonstrated that the compound interacted at sites other than the catalytic sites of BACE1 (Figure 1). Sulforaphane exhibited distinguished suppression against BACE1 activity compared with those of positive controls, quercetin and resveratrol (IC_50_ 18.10 ± 0.03 and 15.04 ± 0.87 μM). Previously published natural BACE1 inhibitors with various structural types, such as catechins, flavonoids, stilbenes, tannins, coumarins, chromones, and others, exhibit inhibitory activity, with IC_50_ values ranging from >200 μM to 1.6 μM [16], which suggests that sulforaphane demonstrates to be a potent BACE1 suppressor with low micromolar potency.

### 3.2. BACE1 Specificity of Sulforaphane

To prevent cross-inhibition side effects, it is important to confirm that sulforaphane is selective over other off-target enzymes. Sulforaphane did not show significant inhibition against serine proteases such as trypsin, chymotrypsin, and elastase that play important roles in various normal physiological processes including digestion, embryonic development, inflammatory responses, and immunity [18]. The compound also exhibited no selectivity against other aspartyl proteases such as BACE2, cathepsin D, and pepsin (Table 2). With aspartic proteases being found in nearly all body tissues and involved in various physiological functions, off-target activity causing severe side effects is possible and likely if an inhibitor is not specific to BACE1 [19]. LY2811376, a promising clinical candidate of BACE1 inhibitor (IC_50_, 0.24 μM), did not proceed to phase II clinical trials because of its off-target activity against cathepsin D [20]. The present results clearly suggest that our compound only suppressed specific targeted enzyme BACE1 without causing adverse effects.

### 3.3. Molecular Docking Simulation of Sulforaphane

**Figure 2:**
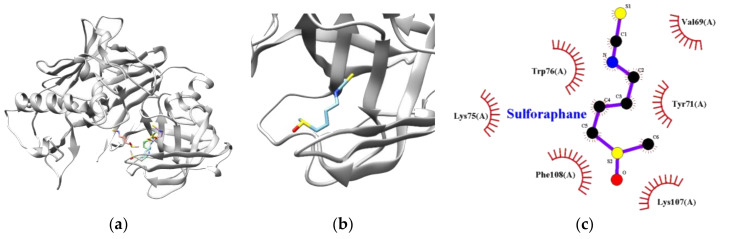
The best docking poses of sulforaphane (**a**) with BACE1. Magnified view of the binding site for ligand–target enzyme complex (**b**). The hydrophobic interaction diagram of BACE1 and sulforaphane (**c**).

For further exploration of the BACE1–sulforaphane interaction, computational docking simulation was employed. As illustrated in Figure 2, the interaction of sulforaphane docked at a non-catalytic site of BACE1, which is consistent with the kinetic studies. BACE1 residues such as VAL69, TYR71, LYS75, TRP76, LYS107, and PHE108 were involved in van der Waals interactions. The BACE1–sulforaphane complex exhibited the lowest energy and the free energy of −3.70 kcal/mol and −6.05 kcal/mol, respectively (Table 3).

According to our present study, sulforaphane, as a non-competitive BACE1 inhibitor, had high selectivity for BACE1 as compared to the other aspartyl and serine proteases, indicating that the compound was a safe natural inhibitor of side effects. Many natural BACE1 inhibitors showed non-competitive modes, such as green tea catechins, stilbenoids, coumarins, citrus flavonoids, and ellagitannin [21,22,23,24]. A previous study by Fang et al. (2017) demonstrated that non-competitive BACE1 inhibitors from natural sources deserved further study because of their non-competitive inhibition modes and different structures from those active site directed inhibitors, which may support a new approach to overcoming problems faced with competitive inhibition [16]. For example, the selectivity of non-competitive BACE1 inhibitors against other aspartyl proteases may be comparatively high, and other roles of BACE1 except Aβ formation may not be suppressed by non-competitive inhibitors, therefore decreasing the adverse effects.

Sulforaphane has received considerable recent attention because of its neuroprotective effect in several previous in vitro and in vivo studies. In vitro studies showed that sulforaphane decreased neuronal toxicity induced by Aβ, tau protein, methylglyoxal, and hydrogen peroxide [25,26,27]. In addition, the compound exerted neuroprotective activity by modulating mitogen-activated protein kinase (MAPK) signaling pathways involved in apoptotic cell death [28]. Administration of sulforaphane ameliorated cerebral vasospasm and early brain injury through nuclear factor erythroid 2-related factor 2 (NRF2) signaling activation, which in turn induced antioxidant enzymes including heme oxygenase-1 (HO-1), gamma-glutamylcysteine synthase (GCS), and glutathione reductase and suppressed inflammatory responses [29,30]. In another study, Bahn et al. revealed that sulforaphane attenuated both Aβ production and cognitive dysfunction through NRF2 activation in an AD mouse model [31].

The blood–brain barrier (BBB) is constituted by neurovascular units that contain endothelial cells advocated by the neuroglia [32]. The restrictive feature of the BBB results in difficulty of molecule delivery to the CNS, and, thus, efforts have mainly been made to create a way to bypass or modulate the BBB for delivery of bioactive compounds [33]. Interestingly, a previous study reported that gavage administration of sulforaphane penetrated BBB in its intact structure and accumulated in brain tissues with a maximum increase and disappearance after 15 min and 2 h, respectively [34].

When consumed, sulforaphane is conjugated with glutathione (GSH) and sequentially metabolized to form several metabolic conjugates including sulforaphane-GSH, sulforaphane- cysteine, and sulforaphane-N-acetylcysteine, which were found in the CNS of mice [35]. Furthermore, sulforaphane was demonstrated to convert to its thioether analog erucin, which is then metabolized in an identical manner to those of sulforaphane [36]. Interestingly, erucin and its metabolites were proven to possess similar neuroprotective properties compared to those of sulforaphane and its metabolites [37].

Sulforaphane from cruciferous vegetables is considered to be safe and nontoxic. Based on the results from toxicity studies, the median toxic dose (TD_50_) and the median lethal dose (LD_50_) of sulforaphane were 191.58 mg/kg and 212.67 mg/kg, respectively. In addition, several clinical studies proved the safety and tolerance of the compound [38,39,40]. Altogether, its ability to penetrate the BBB and its neuroprotective effect without toxicity emphasize the importance of further characterizing sulforaphane’s protective mechanisms of action against AD.

## 4. Conclusions

Although the pathological mechanisms of AD have not been exactly clarified yet, various evidence supports the notion that BACE1 is the rate-limiting enzyme in Aβ production and the critical pathogenic events that lead to AD. The present study revealed that sulforaphane is a novel BACE1 inhibitor with high potency and selectivity which, together with its previously proven safety and good BBB penetration property, could be used as a promising candidate in AD prevention.

## Figures and Tables

**Table 1:** Inhibitory effect and kinetic parameters of sulforaphane against BACE1.

Compound	IC_50_^1^	Concentration (μM)	K_i_^2^	K_m_	V_max_	Inhibition Mode ^3^
Sulforaphane	2.08 ± 0.19	0.03	3.1	3052	3185	Non-competitive
		0.3			2833	
		1.5			2326	
		3			1838	
Quercetin ^4^	18.10 ± 0.03	−	−	−	−	Competitive ^5^
Resveratrol ^4^	15.04 ± 0.87	−	−	−	−	Non-competitive

^1^ The 50% inhibition concentration (µM) of a BACE inhibitor is expressed as mean ± S.D. of triplicate experiments; ^2^ Ki value (µM) represents the binding affinity between the inhibitor and the enzyme; ^3^ Indicates inhibition type of inhibitor determined by Dixon and Lineweaver–Burk plots; ^4^ Quercetin and resveratrol were used as positive control in the BACE1 assay; ^5^ Reference flavonols and flavones as BACE-1 inhibitors: structure–activity relationship in cell-free, cell-based and in silico studies reveal novel pharmacophore features (Shimmyo et al [17]).

**Table 2:** Inhibitory activity (%) ^1^ of sulforaphane against serine and aspartic proteases.

Concentration (μM)		Serine Proteases			Aspartic Proteases			
		Trypsin	Chymotrypsin	Elastase		BACE2	Pepsin	Cathepsin D
Sulforaphane	50	6.01 ± 1.86	12.39 ± 1.96	3.23 ± 0.93		11.25 ± 1.04	4.57 ± 0.25	3.73 ± 0.12
	100	1.40 ± 0.46	14.53 ± 0.74	3.03 ± 0.19		6.11 ± 0.69	5.12 ± 0.30	4.78 ± 0.25

^1^ The inhibitory activity (%) is expressed as mean ± SD.

**Table 3:** Molecular interaction of BACE1 and sulforaphane.

Target Enzyme	Lowest Energy (kcal/mol)	Free Energy (kcal/mol)	Van Der Waals Residues
BACE1	−3.70	−6.05	VAL69, TYR71, LYS75, TRP76 LYS107, PHE108
